# Supplementary material for: Drosophila serotonin 2A receptor signaling coordinates central metabolic processes to modulate aging in response to nutrient choice
Source: eLife. 2021 Jan 19;10:e59399. doi: 10.7554/eLife.59399 (PMC7909950; doi:10.7554/eLife.59399)
Supplement: Supplementary file 1. — Values represent mean lifespan (SEM) in days for each cohort. p-Values are from log-rank ratio test. [file elife-59399-supp1.docx]

| Sex | Strain | Fixed Diet | Choice Diet | %Change | *P*-value |
| --- | --- | --- | --- | --- | --- |
| Male | *Canton-S* | 60.85 (1.48) | 47.23 (1.55) | 28.84% | < 0.001 |
|  | *w*^1118^ _Pletcher lab_ _strain1_ | 59.73 (1.48) | 46.19 (1.65) | 29.31% | < 0.001 |
|  | *w*^1118^ _Pletcher lab_ _strain2_ | 61.59 (1.90) | 44.86 (1.96) | 37.29% | < 0.001 |
| Female | *Canton-S* | 54.71 (0.96) | 52.02 (0.96) | 5.17% | 0.033 |
|  | *w*^1118^ _Pletcher lab_ _strain1_ | 54.56 (1.04) | 51.21 (1.29) | 6.54% | 0.246 |
|  | *w*^1118^ _Pletcher lab_ _strain2_ | 52.48 (0.90) | 45.65 (1.25) | 14.96% | 0.010 |
