# Supplementary material for: Drosophila serotonin 2A receptor signaling coordinates central metabolic processes to modulate aging in response to nutrient choice
Source: eLife. 2021 Jan 19;10:e59399. doi: 10.7554/eLife.59399 (PMC7909950; doi:10.7554/eLife.59399)
Supplement: Supplementary file 2. — RNA-seq data are obtained from FlyAtlas2 (flyatlas.gla.ac.uk/FlyAtlas2/). [file elife-59399-supp2.docx]

|  | **Adult Male** | | **Adult Female** | |
| --- | --- | --- | --- | --- |
| **Tissue** | **FPKM** | **Enrichment** | **FPKM** | **Enrichment** |
| Head | 23 | 4.6 | 17 | 8.7 |
| Eye | 95 | 19 | 72 | 36 |
| Brain / CNS | 16 | 3.1 | 17 | 8.4 |
| Thoracicoabdominal ganglion | 7.9 | 1.6 | 9.3 | 4.7 |
| Crop | 3.3 | 0.7 | 5.7 | 2.9 |
| Midgut | 0.8 | 0.2 | 0.2 | N.A. |
| Hindgut | 0.9 | 0.2 | 0.4 | N.A. |
| Malpighian Tubules | 0.3 | 0.1 | 0.3 | N.A. |
| Salivary gland | 172 | 34 | 138 | 69 |
| Ovary |  |  | 0.1 | N.A. |
| Virgin Spermatheca |  |  | 0.3 | N.A. |
| Mated Spermatheca |  |  | 0.1 | N.A. |
| Testis | 5.0 | 1.0 |  |  |
| Accessory glands | 8.5 | 1.7 |  |  |
| Carcass | 3.1 | 0.6 | 3.2 | 1.6 |
| Rectal pad | 2.2 | 0.4 | 2.2 | 1.1 |
